# PB.21. Is the technical quality of screening mammograms lower in Asian women who cannot understand the English language?

**DOI:** 10.1186/bcr3728

**Published:** 2014-11-03

**Authors:** A Steele, J Morris, A Jain, S Iram, V Reece, M Griffiths

**Affiliations:** 1 The University of Manchester, UK; 2 The Nightingale Centre and Genesis Prevention Centre, Manchester, UK

## Introduction

Good patient and radiographer communication is vital for good quality mammograms. Many Asian women 47 to 73 years old cannot understand English. The Radiographers QA Guidelines categorise mammographic images as Perfect, Good, Moderate and Inadequate (PGMI). The standards required are 75% PG, 97% PGM and <3% I (inadequate).

## Methods

One hundred women with digital screening mammograms were randomly selected retrospectively and divided into two groups: Group A, 50 Asian women who could not understand English (confirmed from radiographer notes on screening forms); Group B, 50 Caucasian women. Any patients with disability, learning difficulties or previous breast surgery were excluded. Two folders were created on PACS and images were anonymised. The films readers were not aware of the classification of the two groups. All mammograms were independently assessed by two experienced film-reader advanced practitioners using the PGMI scoring system. The final score for each case was reached by consensus with a consultant breast radiologist.

## Results

The PGMI mammographic scores for the Asian women are P (0), G (16), M (26), I (8) and scores for the Caucasian women are P (0), G (31), M (18), I (1). The mammographic scores of 32% PG, 84% PGM and 16% I for the Asian women are significantly lower than the scores of 62% PG, 98% PGM and 2% I for the Caucasian women (linear trend test *P *= 0.001).

## Conclusion

The mammographic technical quality appears much lower in Asian women who cannot understand English, highlighting a need for improving communication. Further studies are also required to see whether other factors play any role in the poor technical quality in this group.

